# Physical, chemical and biological properties of MTA Angelus and novel AGM MTA: an in vitro analysis

**DOI:** 10.1186/s12903-025-05517-1

**Published:** 2025-01-27

**Authors:** Sara Nashibi, Parisa Amdjadi, SeyedehSana Ahmadi, Sara Hekmatian, Maryam Torshabi

**Affiliations:** 1 School of Dentistry, Shahid Beheshti University of Medical Sciences, Tehran, Iran; 2 Dental Research Center, Research Institute for Dental Sciences, Shahid Beheshti University of Medical Sciences, Tehran, Iran; 3 Department of Dental Biomaterials, School of Dentistry, Shahid Beheshti University of Medical Sciences, Tehran, Iran

**Keywords:** Calcium ion release, Cytotoxicity, Dental materials, Materials testing, Mineral trioxide aggregate, MTA-Angelus, pH analysis, Radiopacity, Setting time, Toxicity

## Abstract

**Introduction:**

Mineral trioxide aggregate (MTA) is a calcium silicate-based cement that has changed conventional dental therapeutic approaches. This study aimed to evaluate physical, chemical and biological properties of novel AGM MTA, in comparison with MTA Angelus.

**Methods:**

The samples were prepared according to the manufacturer’s instructions. The initial and final setting times were measured via a Gillmore needle following the ISO 6876:2012 standard. The radiopacity of the materials was evaluated against an aluminium step wedge on the basis of the ISO 6876 and 13,116 standards. The pH changes were measured at intervals of 3, 6, 24, 72, 96 and 144 h postimmersion in Hank’s solution and calcium ion release was analysed after 168 h of immersion via inductively coupled plasma optical emission spectroscopy (ICP‒OES). Moreover, the cytotoxicity was assessed through the MTT assay on human dental pulp stem cells (hDPSCs) after 24 and 72 h of exposure to the set/fresh status of various dilutions of MTA extracts, following the ISO 10993-12 standard.

**Results:**

No significant difference was found between the initial setting times of the two materials (Angelus: 11.0 ± 1.0 min, AGM: 10.3 ± 1.5 min); however, MTA Angelus demonstrated a significantly shorter final setting time. Both materials met the minimum radiopacity requirements according to the ISO 6876 standard, with MTA Angelus exhibiting greater radiopacity than AGM MTA. Both materials created an alkaline environment without presenting any differences in each time point and AGM MTA released significantly greater amounts of calcium ions. In the cytotoxicity assessment, while the diluted extracts of both materials did not elicit any cytotoxic effects, the nondiluted samples, after 72 h of exposure, as well as the 30-min set AGM MTA after 24 h of exposure, were shown to be cytotoxic.

**Conclusions:**

In conclusion, MTA Angelus presented a faster setting time and lower cytotoxicity, while AGM MTA demonstrated greater calcium ion release. However, both materials presented clinically acceptable properties and AGM MTA could be a potential alternative to MTA Angelus. However, further clinical studies are required to confirm its application.

## Introduction

The invention of mineral trioxide aggregate (MTA) by Torabinejad in the early 1990s changed the conventional therapeutic approaches employed for various dental treatments [1, 2]. It seems that MTA has been used in various endodontic treatments, including the repair of perforations and root resorption, vital pulp therapy, direct pulp capping, root-end filling, apexification and regenerative endodontic treatments [3]. It has been reported that MTA appears to affect the prognosis of teeth that are considered hopeless [4].

Since the applied materials in the abovementioned endodontic treatments might be in contact with pulp and periapical tissues, the material’s characteristics are of utmost importance [5]. Fundamentally, the biocompatibility of the repair material without stimulation of the tissue inflammatory response is crucial for successful endodontic treatment [6]. Moreover, an ideal restorative endodontic material should exhibit great sealing, dimensional and colour stability, insolubility in contact with body fluids, proper radiopacity, calcium ion release, alkaline pH and bioactivity [7]. The MTA provides most of the expected demands of an ideal endodontic restorative material, providing high biocompatibility and bioactivity [8]. Additionally, MTA seems to participate in periodontal ligament (PDL) and cementum regeneration as well as dentine bridge formation [9].

Although MTA has solved many problems associated with traditional endodontic restorative materials, some drawbacks seem to remain: the presence of toxic elements (e.g., heavy metals and tricalcium aluminate) [10–12], the potential for discolouration due to the presence of various elements (i.e., bismuth, iron, aluminium and magnesium) [13, 14], long setting time, difficult handling and high material costs are some of the issues preventing clinicians from using MTA. This highlights the necessity for further research on novel materials [5, 11, 15].

MTA Angelus (Angelus, Londrina, PR, Brazil) is one of the first commercial MTA products that was introduced in 2001, containing of 80% portland cement and 20% bismuth oxide [16, 17]. This material consists of a powder part (mainly containing tricalcium silicate, dicalcium silicate, tricalcium aluminate, calcium oxide and bismuth oxide) and a liquid part (distilled water) [18]. This restorative material became attractive to clinicians due to a reduction in setting time compared with that of previous products by eliminating calcium sulfate in its composition [2]. The MTA Angelus is known as a biocompatible material, without mutagenic effects and eliciting cytotoxicity [17].

The novel AGM MTA (AGM, Tehran, Iran) is a calcium silicate-based cement that has been introduced most recently, claiming to have calcium ion release, high alkalinity and radiopacity without affecting teeth colour. This material consists of a powder part (mainly containing tricalcium silicate, dicalcium silicate, calcium aluminate and zirconium oxide) and a liquid part (distilled water, calcium chloride, thickening agent) [19].

The composition of both materials is mainly based on tricalcium silicate and dicalcium silicate. However, AGM MTA contains zirconium oxide as the radiopacifying agent instead of bismuth oxide. Zirconium oxide is known as an alternative radiopacifier for MTA materials, nevertheless, bismuth is the main radiopacifier in commercial brands, including MTA Angelus [20]. Furthermore, it has been reported that bismuth oxide might affect the hydration mechanism of MTA and exhibit cytotoxic effects on pulp stem cells, without promoting cell growth [21, 22]. Moreover, it has been revealed that the addition of zirconium oxide to calcium silicate materials resulted in improved biocompatibility [23, 24]. Moreover, the liquid composition in AGM MTA, which in addition to distilled water contains calcium chloride and a thickening agent, is different from MTA Angelus. The studies revealed that addition of thickening agent to the distilled water into the MTA composition could improve its handling [25].

The different composition of MTA Angelus and AGM MTA might result in different physical, chemical and biological properties. A recent study investigated the antibacterial and antifungal properties of AGM MTA compared to Ortho MTA, ProRoot MTA and Cem cement [26]. However, to the best of the authors’ knowledge, no previously published studies investigating the setting time, radiopacity, pH, calcium ion release and cytotoxicity of this endodontic material were found in the literature. Consequently, the current study aimed to evaluate and compare the physical, chemical and biological properties of the novel AGM MTA with the MTA Angelus. The findings of these experiments could increase the knowledge of dental endodontic materials and how the altered chemical composition of an endodontic material results in different properties. Furthermore, the evaluation of recently introduced materials is necessary to provide enough information for attaining an ideal endodontic material, which overcomes previous limitations. The null hypothesis is that AGM MTA does not significantly differ from MTA Angelus in terms of the abovementioned physical, chemical and biological properties.

## Methods and materials

### Material preparation and setting time

Prior to the beginning of the experiments, all the apparatus were conditioned for 24 h under 98% relative humidity at 37 ± 1 °C in an incubator (Pars Azma Co., Tehran, Iran). The materials used in the present study were MTA Angelus and AGM MTA (Table 1) [19, 27]. The cements were mixed on a plate of glass based on the manufacturers’ recommended instructions to reach a homogeneous mixture. The mixed experimental materials were filled into a mould with an internal diameter of 10 mm and a height of 1 mm [28, 29]. Three discs (*n* = 3) were prepared for each group [30]. The setting time test was performed according to the American Society for Testing and Materials specifications (ASTM C266-08), International Organization for Standardization (ISO) 6876 standard and the method previously reported by T.-Y. Kang et al. [30]. The analysis was performed at a temperature of 37 ± 1 °C and under 98% relative humidity. Initial and final setting time tests were evaluated using two Gillmore needles (Humbold MFG Co., Norridge, IL, USA) with masses of 113.4 g and 453.6 g, respectively. The Gillmore needles were placed vertically on the specimens every 60 s until no indentation was observed. The same preparation method was performed for radiopacity, pH variation and calcium ion release analyses.

**Table 1:** Information on the two experimental cements (AGM MTA and MTA angelus) used in the present study

Materials	Composition	Manufacturer	Batch Number
**AGM MTA**	Powder: tricalcium silicate, dicalcium silicate, calcium aluminate, zirconium oxide, etc. Liquid: distilled water, calcium chloride, thickening agent	AGM, Tehran, Iran	01040205
**MTA Angelus**	Powder: tricalcium silicate, dicalcium silicate, tricalcium aluminate, calcium oxide, bismuth oxide, etc. Liquid: distilled water	Angelus, Londrina, PR, Brazil	41,822

## Radiopacity

The radiopacity test was performed according to the ISO 6876 and 13,116. The prepared moulds were placed in an incubator with 98% relative humidity at 37 ± 1 °C to allow for complete setting. Afterwards, the moulds were removed and each MTA disc was placed on a digital sensor (Digora, Soredex, Tuusula, Finland) along with an aluminium step wedge (made from 99.5% pure aluminium, graduating from 1 to 10 mm) [31]. Radiographic images were obtained using a dental X-ray unit (Carestream, CS2100, Atlanta, GA, USA) operating at 65 ± 5 kV and 7 mA with a 0.3 s exposure time and 30 cm focus‒film distance [32]. The average grey value pixels of each MTA disc, as well as each step of the aluminium step wedge, were determined in Adobe Photoshop (Adobe, San Jose, CA, USA), using five points randomly selected on the specimen, regardless of the presence of air bubbles [33, 34]. The equivalent radiopacity of each specimen was calculated in millimitre of aluminium on the basis of the approach of M. A. H. Duarte et al. [35]. Moreover, an alternative method was designed to enhance the clarity of this approach, which led to similar results. In this manner, a graph was created through Microsoft Excel version 16.0 (Microsoft Corp., Redmond, Washington, United States) to demonstrate the mean pixels of each step of the aluminium step wedge. This graph served as a reference to determine the equivalent grey level of the experimental materials in millimetres of aluminium.

## pH variation and calcium ion release

The prepared specimens were placed in an incubator at 37 ± 1 °C and 98% relative humidity for 24 h to set completely. After the separation of the moulds, each MTA disc was immersed in 10 mL of Hank’s balanced salt solution (HBSS Gibco, Paisley, UK). The pH values were evaluated via Jenway 3310 pH meter (Jenway, Essex, UK), which was calibrated with buffer solutions with pH values of 4.01, 7.00 and 10.01. The pH changes were measured at 3, 6, 24, 72, 96 and 144 h after the immersion of the specimens in HBSS. Between the measurements, the solutions were stored in an incubator under the abovementioned conditions. Moreover, the electrode was washed with distilled water and dried with a specific tissue after each measurement [30].

The calcium ion release test was performed using the same solutions used for the pH analysis. After 168 h of immersion of the specimens in HBSS, the solutions were filtered through a 0.22 μm syringe filter (Membrane Solutions, Washington, USA). Afterwards, the calcium ion concentrations were measured via ICP-OES Spectro analaytical instruments (Spectro Arcos, Kleve, Germany) [30].

## Cytotoxicity

### Material preparation

Each MTA material was placed in cylindrical moulds with a diameter of 7 mm and a height of 2 mm in a sterile environment. The specimens were divided into two groups: Group A consisted of materials with 30 min of setting and Group B consisted of materials that had 24 h to set. Three discs (*n* = 3) were prepared for each time point of each material. The specimens were stored in an incubator at 37 °C and 98% relative humidity during the experiment. The extraction was subsequently performed according to ISO 10993-12 standards via high-glucose Dulbecco’s modified Eagle’s medium (DMEM; Gibco, Paisley, UK) supplemented with 10% fetal bovine serum (FBS; Gibco, Paisley, UK) in an incubator as described above. All specimens were placed into the wells of 24-well plates and immersed in DMEM with an approximate surface area of 3 cm² per mL of medium. All the specimens, in addition to those in the control group (culture medium containing serum without the materials), were incubated for 24 h. After 24 h of incubation, the extractions were sterilised using a 0.22 μm syringe filter (Membrane Solutions, Washington, USA) and collected in sterile microtubes [36].

### MTT assay

Human pulp dental stem cells (DPS-1, Iranian Biological Resource Center: IBRC: C10265, Tehran, Iran) at the fifth passage were cultured in high-glucose DMEM supplemented with 10% FBS, 100 µg/mL streptomycin and 100 IU/mL penicillin (Gibco, Paisley UK). The specimens were stored in an incubator with 5% CO2 and 95% humidity at 37 °C (Binder, Tuttlingen, Germany).

To evaluate cytotoxicity via the extraction method (indirect exposure), a 3-(4,5-dimethylthiazol-2-yl)-2,5-diphenyltetrazolium bromide (MTT) assay was performed according to ISO 10993-5:2009 and ISO 10993-12:2021. The cells were counted after adherent cells were detached during their logarithmic growth phase via a mixture of trypsin/ethylenediaminetetraacetic acid (Gibco, Paisley UK). The hDPSCs were cultured in a 96-well plate at a density of 8000 cells/well in complete medium. Afterwards, the plates were incubated in the cell culture incubator for 24 h to reach exponential cell growth. After 24 h of culture, the culture media were removed and replaced with 100 µl/well extracts [undiluted (100%) and diluted (50%, 25%)] of the tested materials or control media. After 24 h (for acute cytotoxicity assessment) and 72 h (for chronic cytotoxicity assessment) of incubation, the plates were removed from the incubator and digital images were captured using an inverted light microscope (Nikon, Tokyo, Japan). The medium in each well was subsequently replaced with 100 µL of culture medium containing MTT. The plates were then incubated for 3 h until formazan crystals formed. Following the evacuation of the MTT solution from each well, 100 µL of dimethyl sulfoxide (DMSO; Sigma-Aldrich, Taufkirchen, Germany) was added to dissolve the formazan crystals. Finally, the optical density (OD) of the solutions in each well was read via an ELISA reader (Anthos 2020, Salzburg, Austria) to calculate cell viability.

### Statistical analysis

The results of the experiments were analysed via GraphPad Prism version 9 (GraphPad Prism Software, Inc. La Jolla, CA, USA). To analyse the significant differences between the two experimental groups, a t-test analysis was performed for setting time, radiopacity, pH variation and calcium ion release. Furthermore, one-way ANOVA followed by Tukey’s *post hoc* test was used for cytotoxicity assessment. P values less than and/or equal to 0.05 were considered statistically significant.

## Results

### Setting time

Table 2 Presents the means, standard deviations and p-values of the initial and final setting time analyses of the experimental materials. While no significant difference was observed between the initial setting times of AGM MTA and MTA Angelus, MTA Angelus suggested a shorter setting time compared to AGM MTA.

**Table 2:** Initial and final setting times of AGM MTA and MTA angelus (mean ± standard deviation)

Materials	Setting time (min)	
	Initial setting time	Final setting time
**AGM MTA**	11.0 ± 1.0	50.7 ± 1.2
**MTA Angelus**	10.3 ± 1.5	18.0 ± 1.0
***P *** **value**	**0.5614**	**< 0.0001**

### Radiopacity

**Fig. 1:**
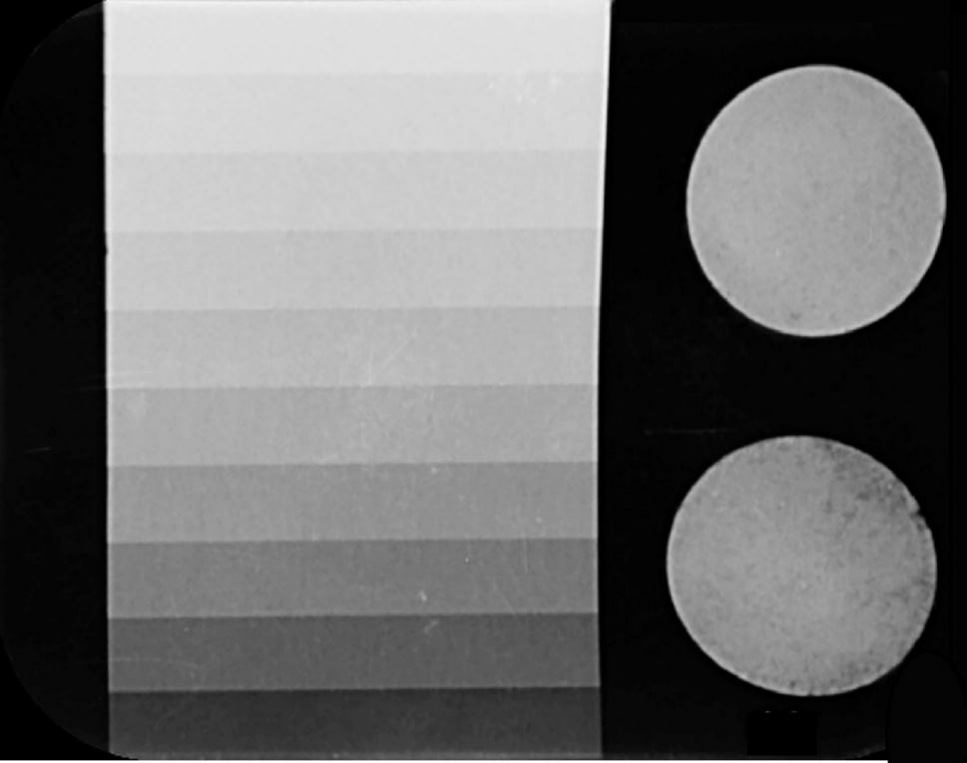
Radiographic image of AGM MTA (the bottom specimen) and MTA Angelus (the upper specimen) along with the aluminium step wedge

**Fig. 2:**
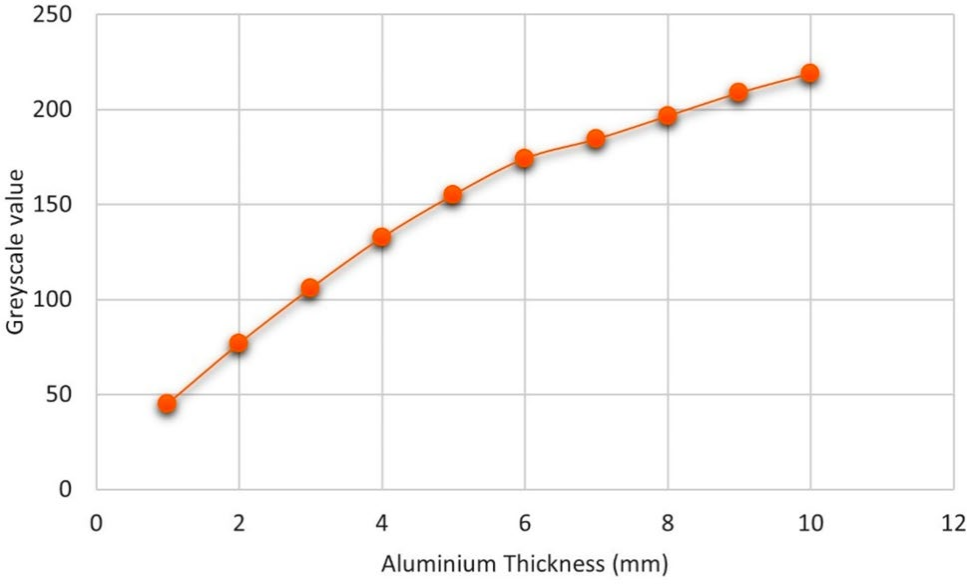
Graph of grey pixel values ​​versus aluminium thickness (mm)

Figure 1 shows radiographic image of the specimens along with the aluminium step wedge. In addition, the graph of the average grey pixels of each aluminium step is shown in Fig. 2. The means, standard deviations and p-values of the radiopacity of the tested materials are presented in Table 3. Compared to AGM MTA, MTA Angelus was more radiopaque. Nevertheless, both materials achieved the minimum required radiopacity of 3 mm aluminium, according to ISO 6876 [37].

**Table 3:** Radiopacity of AGM MTA and MTA Angelus (mean ± standard deviation)

Materials	Radiopacity (mm Al)
**AGM MTA**	4.1 ± 0.44
**MTA Angelus**	4.84 ± 0.23
***P *** **value**	**0.0105**

### pH variation and calcium ion release

The means, standard deviations and p-values of pH and calcium ion release of AGM MTA and MTA Angelus at the specified time intervals, are presented in Table 4. Both materials created an alkaline environment across different time intervals. According to the statistical analysis of pH variations, AGM MTA and MTA Angelus presented similar results in each time point. However, the calcium ion release of AGM MTA was significantly greater than that of MTA Angelus after 168 h.

**Table 4:** pH variation and calcium ion release (mg/L) in HBSS for 7 days (mean ± standard deviation)

Materials	pH						Ca Ions concentration (mg/L)
	3 h	6 h	24 h	72 h	96 h	144 h	168 h
**AGM MTA**	12.25 ± 0.02	12.52 ± 0.00	12.42 ± 0.01	12.85 ± 0.05	13.00 ± 0.07	12.56 ± 0.07	888.0 ± 52.4
**MTA Angelus**	12.35 ± 0.03	12.56 ± 0.04	12.53 ± 0.05	12.92 ± 0.03	13.05 ± 0.03	12.77 ± 0.08	667.7 ± 3.5
***P*** **value (AGM vs. Angelus)**	**0.2857**	**0.1448**	**0.174**	**0.0961**	**0.2587**	**0.1254**	**0.0019**

### Cell cytotoxicity

**Fig. 3:**
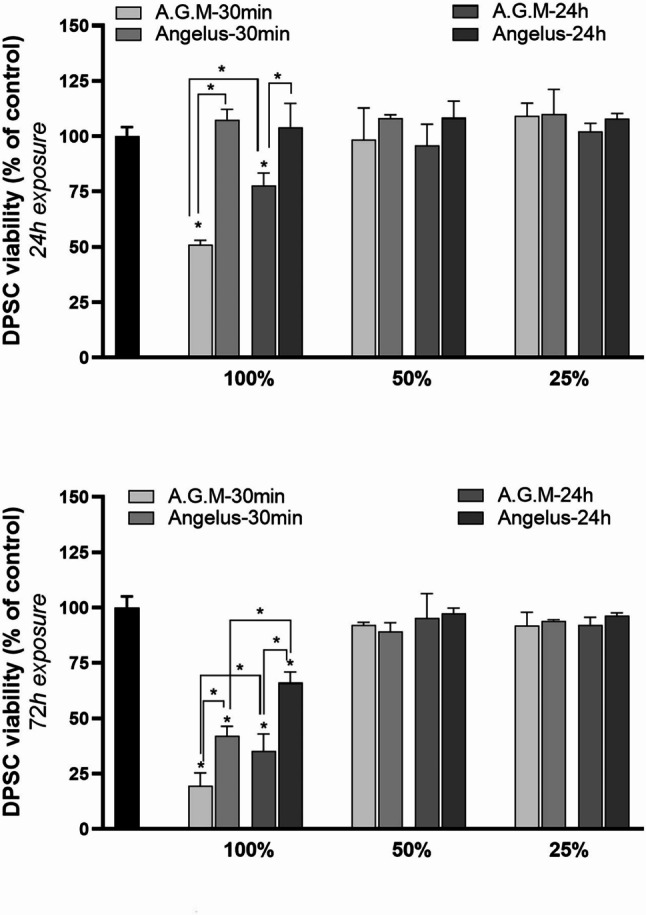
Graphs present the viability (%) of hDPSCs in contact with various concentrations of cement extracts (100%, 50%, 25%) after 24 and 72 h of exposure in comparison with the control group

The cell cytotoxicity of different dilutions of fresh/set material extracts on hDPSCs after 24 h and 72 h of exposure is illustrated in Fig. 3. The diluted extracts (50%, 25%) were associated with increased cell viability, indicating that neither MTA materials elicited any cytotoxic effects after 24 h or72 h of exposure. Considering the state of the materials, cements with a 24-hour setting time exhibited greater cell viability compared to 30-minute set materials. Furthermore, in the 24-hour culture, AGM MTA with a 30-minute setting time was found to be cytotoxic (under 70% cell viability) despite the 24-hour set material. Moreover, all the materials demonstrated greater cytotoxicity after 72 h of exposure compared to 24 h. Despite the biocompatibility of MTA Angelus in the 24-hour culture, this cement demonstrated cytotoxic effects in both the fresh and set states after 72 h of culture. Eventually, undiluted (100%) extracts of AGM MTA were significantly more cytotoxic than MTA Angelus without considering other variables.

**Fig. 4:**
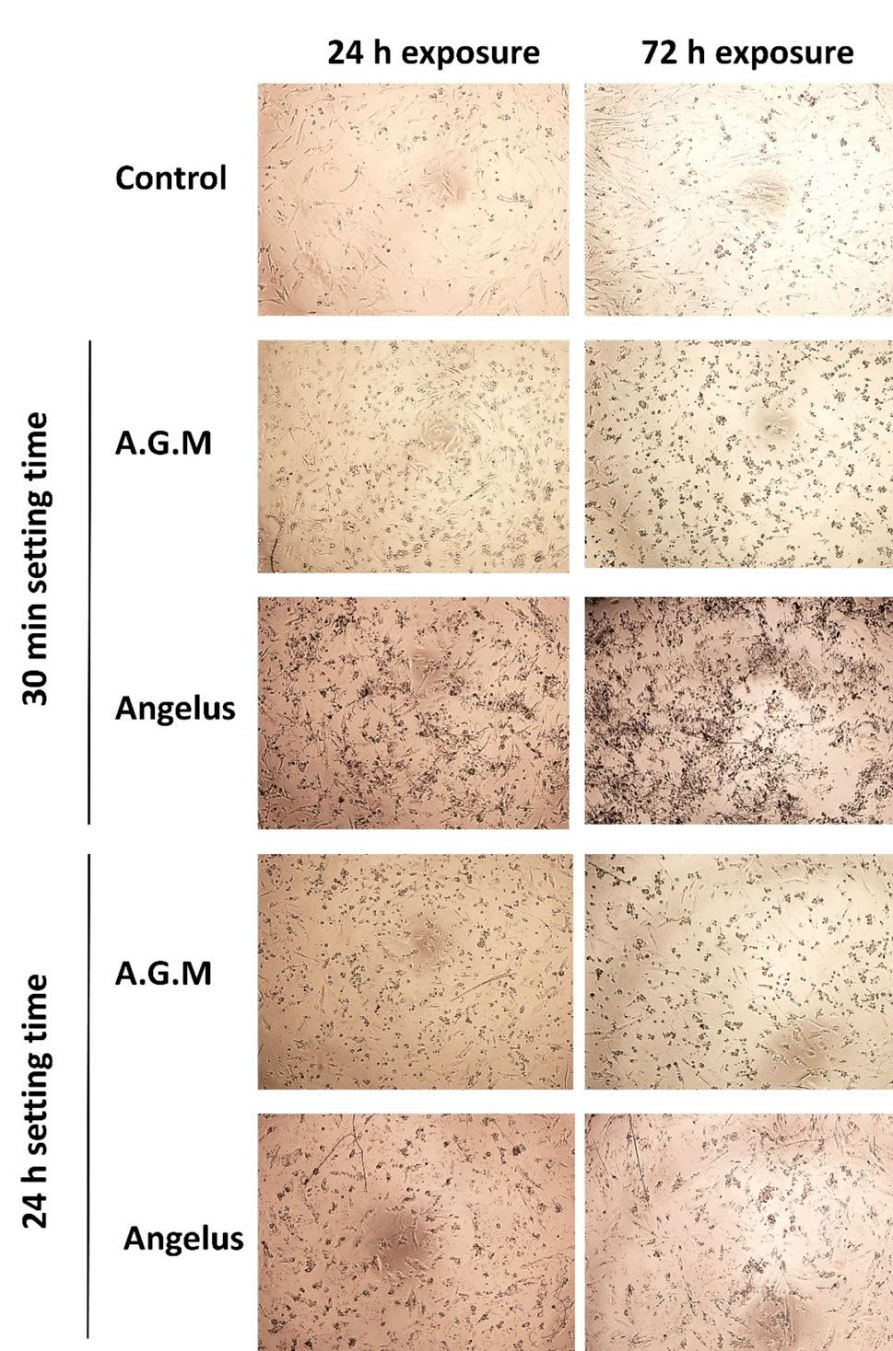
Microscopic view (magnification 40×) of the morphology of hDPSCs

The images obtained from the inverted light microscope are shown in Fig. 4. The AGM cement group with a 30-minute setting time, after both 24 h and 72 h of exposure, exhibited a round cell morphology, indicating damaged cells. However, the other groups presented healthy cells with a spindle-shaped and elongated morphology.

## Discussion

Conventional materials and techniques owe their massive transformation to increased patient demand for tooth preservation and new cement technologies that have emerged over the past few decades. Calcium hydroxide has been considered a standard material used in a wide range of treatments, from indirect pulp capping to apexification. However, its drawbacks have led to some compositional alterations in the hope of improving its clinical efficacy. Ultimately, MTA has emerged as a powerful, versatile and promising endodontic material that can solve conventional complications and preserve teeth in vulnerable clinical conditions [38, 39]. Consequently, the introduction of new endodontic materials with desirable properties to overcome the challenges of conventional MTA cements is of utmost importance. In 2024, F. A. Yalin et al. reported the antibacterial and antifungal effects of AGM MTA along with Ortho MTA, ProRoot MTA and Cem cement [26]. However no previous study has investigated the physical, chemical and biological properties of AGM MTA in comparison with conventional materials.

The physical and chemical properties of a dental material are highly valuable, as they can serve as evidence for its clinical use [40]. No specific standard-setting time exists; however, the setting time of an endodontic material should be long enough to allow manipulation and placement inside the tooth without any gaps or displacement. Furthermore, a long setting time would not be appropriate for clinical use, as it would be dificult to maintain an isolated environment in addition to the possibility of contamination or cement washout [41]. In the present study, although both MTA materials exhibited the same initial setting times (AGM: 11.0 ± 1.0 min, Angelus: 10.3 ± 1.5 min), the final setting time of MTA Angelus was notably shorter (AGM: 50.7 ± 1.2, Angelus: 18.0 ± 1.0 min).

Tricalcium aluminate, which is the most reactive constituent of portland cement, is present in the composition of MTA Angelus. The hydration reaction of this component is faster than tricalcium silicate and participates in the formation of calcium hydroaluminate and calcium hydroaluminate-ferrite phases. The presence of this material is accompanied by a reduction in the setting time, according to its high reactivity and fast hydration reaction [42, 43]. The AGM MTA replaced the bismuth oxide by zirconium oxide as the radiopacifying agent. Previous studies declared that bismuth oxide participates in the formation of calcium silicate bismuth hydrate (C-S-H-Bi) and is a part of the hydrated phase. Additionally, it affects the precipitation of Ca(OH)2 in the hydrated material, which could affect the material’s bioactivity [44]. Several studies have shown the longer setting time of materials with bismuth oxide, as compared to the materials without this agent [44–46]. It was described that the slower hydration reaction and longer setting time in the materials containing bismuth oxide is due to the decreased amount of C-S-H in the hydrated materials [47]. However, conflicting results obtained from the study by M. Martínez-Hernández et al. in 2023, which presented similar setting times among MTA cements with different concentrations of bismuth oxide [48]. In contrast with bismuth oxide, zirconium oxide is an inert radiopacifying agent, which does not participate in reaction with calcium silicate-based cements. This radiopacifyer is found in the composition of AH plus endodontic sealer and the replacement of bismuth oxide with zirconium oxide presented no effect on the setting time of calcium cilicate-based cements [49–51]. The addition of calcium chloride as a hydration accelerator to the MTA composition improves the material’s setting time by promoting development of hydration products in the setting reaction. According to a meta-analysis on relevant in-vitro studies by B. Bolhari et al. in 2024, additive calcium chloride (5% and 10%) in white MTA composition led to a decreased initial and final setting time [52, 53].

Biodentine contains some ingredients similar to those of AGM MTA (e.g. zirconium oxide as the radiopacifier and calcium chloride in its liquid). The study by V. Kharabe et al. in 2023, presented a longer setting time for Biodentine compared to MTA Angelus (Biodentine: 29.2 ± 3.84 min, Angelus: 15.2 ± 2.11 min), which is in agreement with the observed longer final setting time of AGM MTA, as compared to MTA Angelus in the present study. The conflicting results regarding the materials’ setting times, might be related to the variations in powder/liquid ratios, particle sizes and shapes, compositions and the ambient temperature [50, 54, 55]. Moreover, the longer setting time of Biodentine could be due to the different powder‒liquid ratios and the indeterminate percentage of calcium chloride in its mixture [56].

Radiopacity is one of the essential characteristics of materials used for pulp therapies [57]. The contrast of the radiopaque material in radiographic images makes it distinguishable from surrounding tissues, providing the clinician with the opportunity to evaluate the quality of treatment before the final restoration and to avoid treatment failure [58]. In the present study, although MTA Angelus exhibited greater radiopacity than AGM MTA, both materials reached the minimum required level of radiopacity for root canal sealing materials—3 mm—according to ISO 6876 [37]. The composition of AGM MTA contains calcium chloride in its liquid and zirconium oxide as its radiopacifying agent. It has been reported that replacement of saline water with 10% calcium chloride solution in a MTA-like cement resulted in decreased radiopacity from 6.22 ± 0.38 mmAl to 4.10 ± 0.23 mmAl [59]. Moreover, in 2020, M.-S. Chen et al. presented that the addition of zirconium oxide to MTA composition might be accompanied by a decrease in radiopacity due to the lower radiodensity of zirconium oxide compared to bismuth oxide [60]. Additionally, based on reports by Sarunket C et al. in 2022, bismuth oxide is a highly radiopaque radiopacifier in comparison to other materials, including zirconium oxide [61]. Furthermore, in 2023, H. G. Sen et al. demonstrated greater radiopacity for ProRoot MTA compared to Biodentine, which might be associated with the presence of bismuth oxide as the radiopacifier in the composition of ProRoot instead of zirconium oxide. These findings are consistent with the results of our study, proving that the lower radiopacity of zirconia, owing to its lower atomic number, can lead to a lower radiopacity of the material [33].

Previous studies have shown that MTA is capable of encouraging hard tissue formation due to its alkaline activity and the release of calcium ions. An alkaline environment is crucial for the promotion of the healing of dental tissues and remineralisation. Furthermore, a bioactive endodontic material can be recognised based on its ability to release calcium ions and maintain an alkaline environment [62]. The 50–75 wt% MTA composition belongs to calcium oxide [11]. Calcium oxide in an aqueous solution transforms into calcium hydroxide, which further decomposes into calcium and hydroxyl ions [63]. Calcium hydroxide is a product of the hydration reaction of MTA, which can explain the highly alkaline environment surrounding MTA. Moreover, this chemical product reacts with phosphate and leads to the formation of hydroxyapatite, which can act as a biological seal [64].

The necessity of an alkaline environment has been proven because of its increasing effect on antibacterial ability, biocompatibility and osteogenic potential [65]. In the present study, both materials created a highly alkaline environment in the range of 12–13, without any considerable difference. Studies have reported that a pH over 12 is considered inhibitory for most microorganisms. In our study, the pH of two materials slightly decreased at the end of 144 h. However, both materials maintained their alkalinity within a range of pH values greater than 11. These findings are consistent with the study of M. Fridland et al. in 2005, which concluded that MTA is able to release its soluble fraction over a long period of time at a decreasing rate. However, the pH was maintained in the range of 11–12 during the study [66].

Several studies have explained the critical role of calcium ions in promoting the initiation of mineralisation and hard tissue formation. Moreover, the released calcium ions have been shown to diminish the activity of osteoclasts, activate the formation of fibroblasts and increase alkaline activity [67, 68]. Calcium ions are known to be the predominant ions released during the MTA setting reaction [62]. The release of calcium ions relies upon the composition of the cement mineral particles, which are responsible for their solubility and diffusion in water [69]. In the present study, the cements were immersed in HBSS, which is a proper simulation of body fluids. The release of calcium ions was evaluated via ICP‒OES analysis. The results demonstrated greater calcium ion release in AGM MTA after 168 h of immersion compared to MTA Angelus. In 2018, I. Aprillia et al. reported that Biodentine is capable of releasing more calcium ions than MTA Angelus [70]. In addition, according to the study by A. C. R. Rocha et al. in 2015, Biodentine contains calcium chloride in its composition, as opposed to MTA Angelus [71]. Moreover, the study by E. Antunes Bortoluzzi et al. in 2006 mentioned the possible increasing effect of calcium chloride on calcium ion release by MTA [72]. As a consequence, the various amounts of calcium ions released could be attributed to the presence of calcium chloride in the fluid composition of the AGM MTA.

Biocompatibility is known to be one of the most critical properties of materials used for vital endodontic treatments because of their long contact with the surrounding pulp tissue. High biocompatibility is associated with new cementum formation in periradicular tissues, as well as bridge development in the pulp space, which results in the stimulation of favourable reparative responses. In addition, damage or irritation can lead to periapical tissue degeneration and a delay in the wound healing process, highlighting the importance of assessing the cytotoxicity of materials used for endodontic treatments [73, 74]. In vitro assessments are employed for the initial evaluation of new dental materials before their clinical application. In the present study, the cytotoxicity of a new formulation of MTA, named AGM, was evaluated in comparison with MTA Angelus through in vitro analysis. The MTT assay was performed to assess cytotoxicity, which is a measure of cell metabolic activity [36]. Furthermore, hDPSCs were chosen to simulate the oral environment properly.

In this study, both MTA materials showed increased cytotoxicity with longer exposure times (72 h) compared to 24 h of exposure, confirming that the cytotoxicity is time dependent. Moreover, a study by E. A. Koulaouzidou et al. on the cytotoxicity of two MTA brands on human lung fibroblasts and rat pulp cells reported greater cytotoxic effects of all experimental materials after 72 h than after 24 h of exposure [75]. These findings confirm the time dependency of the cytotoxicity of these materials.

In the present study, both fresh and set states of the materials were included in the experiment to determine whether the cytotoxicity is related to the set material or is a result of byproducts released during the setting process [36]. Our results revealed a noticeable difference between the fresh and set states of the same material, as the fresh cement exhibited a greater cytotoxic effect, except for MTA Angelus after 24 h of exposure, where no significant difference was observed between the set and fresh states. These findings are in accordance with the study by K. Keiser et al., which revealed greater cytotoxicity in fresh cements compared to 24-hour set materials in an MTT assay [76].

To assess the effects of dilution on material cytotoxicity, different concentrations of the experimental materials (100%, 50% and 25%) were evaluated via the MTT assay. Regarding the MTAs’ concentrations, the diluted samples of both materials (50% and 25%) did not elicit any cytotoxic effects, whereas contact with 100% extracts could cause high cytotoxicity. This finding provides evidence that the cytotoxicity is dependent on the dose of used materials. Moreover, the effects of exposure time and the different states of MTA materials on cytotoxicity could only be observed in nondiluted samples. These results are consistent with the study by S.-Y. Kim et al., who reported the cytotoxic effects of MTA materials on mesenchymal stem cells (MSCs) at 100% and 50% concentrations, in contrast with more diluted extracts at 25% and 12.5% concentrations [77]. In our analysis, both MTA Angelus and AGM MTA after 72 h of exposure and the 30-min set AGM MTA after 24 h of exposure manifested as cytotoxic cements at a 100% concentration. However, it has been observed that the elution of endodontic cements begins at the moment after their application in a clinical environment. As a consequence, the diluted extracts might provide a more accurate simulation of clinical conditions [78].

Recent studies reported that direct and indirect contact with materials containing bismuth are accompanied by reduced cell viability. Additionally, bismuth release from the cement was observed for a long period of time (i.e., 180 days) and could be detected systemically in kidney, blood, liver and brain. It has been found that the kidney was the organ of choice for the bismuth accumulation [79, 80]. However, several studies indicated that the presence of bismuth oxide as the radiopacifying agent in calcium-silicate based cements does not elicite cytotoxic effects, depending on its concentration [20, 81, 82]. Nonetheless, it has been claimed that the less biocompatibility of MTA in high concentrations might be related to the release of bismuth [83]. Various studies demonstrated low cytotoxic effects of calcium silicate-based cements containing zirconium oxide [81, 84] and similar biocompatibility with bismuth oxide-containing materials [85]. Moreover, it has been reported that zirconium oxide might present more viability, as compared to bismuth oxide [51, 86]. However, it has been reported that zirconium might cause oxidative stress following the accumulation in brain, heart, lungs, spleen, liver and kidney. Furthermore, the damaging effect of zirconia depends on its dosage and further research is required on its long-term toxicity [87, 88].

The caclium chloride in the liquid of Biodentine leads to a rapid release of calcium hydoxide and decreased cell viability after 24 h of exposure. Subsequently, the cytotoxicity of Biodentine might be attributed to the presence of calcium chloride in its liquid [83]. In the present study, 100% extracts of MTA Angelus exhibited higher cell viability compared to AGM MTA and diluted samples of both cements were associated with high viability of hDPSCs without any significant difference. In the study by A. d. P. Melo et al. in 2023, 1:1 dilutions of calcium silicate-based sealers were accompanied by a significant reduction in cell viability. Moreover, it has been pointed out that biological properties of calcium silicate-based cements depend on material’s composition, the ratio of each component and the surface structure, which could explain the different biocompatibility of the materials tested in the present study [89].

### Limitations

The present study has a number of limitations including lack of elemental characterization of the experimental materials, short-term pH analysis, lack of microbial analysis and not performing solubility assay. Future studies should perform comprehensive research on materials’ physical, chemical and biological properties, considering elemental characterization of tested materials. The characterization provides a more detailed understanding of material’s composition, allowing them to correlate the material’s properties with its components. In addition, this analysis could be used as a confirmation of manufacturer reported composition. Moreover, considering the similarities between AGM MTA and Biodentine, future studies could focus on comparing these cements.

## Conclusion

In conclusion, MTA Angelus presented a faster setting time and lower cytotoxicity, while AGM MTA demonstrated greater calcium ion release. However, both materials presented clinically acceptable properties and AGM MTA could be a potential alternative to MTA Angelus. However, further clinical studies are required to confirm its application.

## Data Availability

The datasets used and/or analysed during the current study are available from the corresponding author upon reasonable request.

## Abbreviations

MTA: Mineral trioxide aggregate
ICP‒OES: Inductively Coupled Plasma Optical Emission Spectroscopy
HDPSCs: Human dental pulp stem cells
ISO: International Organization for Standardization
HBSS: Hank’s balanced salt solution
DMEM: Dulbecco’s modified Eagle’s medium
MTT: 3-(4,5-dimethylthiazol-2-yl)-2,5-diphenyltetrazolium bromide
DMSO: Dimethyl sulfoxide
OD: Optical density
MSCs: Mesenchymal Stem Cells
